# A Physics-Informed ConvFormer with State-Space Gating for Smartphone-Based Human Activity Recognition on the WISDM Dataset

**DOI:** 10.3390/s26196139

**Published:** 2026-09-28

**Authors:** Yancheng Pan, Zixin Zhao, Yingwei Xu

**Affiliations:** 1 School of Physical Education, Yanshan University, Qinhuangdao 066000, China; pyczzx2099@stumail.ysu.edu.cn; 2 Department of Physical Education, Southeast University, Nanjing 211189, China; 220243223@seu.edu.cn

**Keywords:** human activity recognition, WISDM, smartphone accelerometer, depthwise convolution, ConvFormer, state-space model, Mamba, edge AI, time series classification

## Abstract

**Highlights:**

**What are the main findings?**

- The proposed Physics-Informed ConvFormer integrates coordinate decoupling, local depthwise convolution, State-Space Gating, and residual attention for WISDM HAR.
- Across four subject-level splits, the full model achieved 88.86 ± 6.16% mean test accuracy, while the compact CNN baseline achieved 89.66 ± 5.56%; this controlled comparison quantifies the accuracy-efficiency trade-off of the proposed hybrid architecture.

**What are the implications of the main findings?**

- Subject-level evaluation reveals a stricter and more realistic cross-user generalization challenge than random window splitting.
- Local convolution and attention provided the strongest contributions within the hybrid backbone, while the raw-input and no-SSM variants identify practical directions for simplifying the model in latency-sensitive applications.

**Abstract:**

Smartphone-based human activity recognition (HAR) requires discriminative temporal modeling and careful assessment of computational cost. This study evaluates a Physics-Informed ConvFormer with State-Space Gating for six-class HAR on the WISDM v1.1 accelerometer dataset. The model combines raw acceleration with deterministic magnitude, directional, gravity-estimate, and dynamic-acceleration features, followed by large-kernel depthwise convolution, a Mamba-inspired causal gate, attention, and a multilayer perceptron. The main subject-level experiment achieved 95.63% best validation accuracy and 83.92% test accuracy. Across four subject-level splits, the full model achieved 88.86 ± 6.16% mean test accuracy, compared with 89.66 ± 5.56% for the compact CNN baseline and 89.54 ± 5.07% for the raw three-channel hybrid variant. The full model used 588,006 parameters and had a measured local CPU forward-pass latency of 21.54 ms per window, whereas the CNN used 125,766 parameters and 0.45 ms. Removing State-Space Gating changed mean accuracy by 0.22 percentage points and reduced latency to 3.80 ms. Removing local convolution or attention reduced mean accuracy by 3.99 or 3.06 percentage points, respectively, within the hybrid backbone. These results show that local convolution and attention are valuable within the hybrid design, while raw-input and no-SSM configurations remain competitive lower-cost alternatives. The study provides a transparent component-level assessment under subject-level separation and defines priorities for future smartphone deployment, orientation-robust evaluation, and longer-context modeling.

## 1. Introduction

Human activity recognition (HAR) using wearable and smartphone inertial sensors is a central problem in mobile health, elder-care monitoring, fitness tracking, digital behavior analysis, and human–computer interaction [1]. Comprehensive surveys further show that wearable inertial sensing has become an important foundation for mobile and ubiquitous HAR systems [2]. Compared with camera-based HAR, inertial sensing is inexpensive, available in everyday devices, and generally less privacy-intrusive because it does not capture identifiable visual scenes; however, accelerometer signals are noisy, orientation-sensitive, highly user-dependent, and frequently imbalanced across activity classes [3,4,5]. The WISDM benchmark originates from the smartphone accelerometer study of Kwapisz et al. [1]. The public data file analyzed here contains more than one million tri-axial samples with 36 user identifiers and six activity labels: Walking, Jogging, Upstairs, Downstairs, Sitting, and Standing.

Deep learning has shifted HAR from handcrafted feature engineering toward end-to-end feature learning. Classical one-dimensional convolutional neural networks (1D-CNNs) learn local temporal motifs from raw sensor streams and have shown strong performance on WISDM [6,7]. Recurrent models and CNN-LSTM hybrids introduce temporal memory but may be harder to deploy efficiently on low-resource devices [8,9]. Transformer-based models further improve global sequence modeling through attention, but the quadratic cost of self-attention and its weaker built-in bias for adjacent time samples can be unattractive for mobile inference [10,11]. Recent selective state-space models, particularly S4 and Mamba-like architectures, provide a compelling alternative because they model long temporal context with linear sequence complexity [12,13,14]. BabyMamba-HAR demonstrates that selective state-space modeling can be compressed into TinyML-scale HAR systems, achieving competitive cross-dataset F1 scores with approximately 27K parameters and practical embedded deployment on microcontrollers [15].

The WISDM problem is useful because it links sensor physics, robust signal representation, and deployable artificial intelligence. A model that performs well only under random window splitting may be less informative for real use than a model whose strengths and weaknesses are reported under subject-level separation. This manuscript therefore treats HAR not only as a classification task but also as a sensor-modeling problem in which physical invariance, temporal locality, and computational cost must be discussed together [3,4,5,16].

At the same time, recent WISDM-focused studies show that strong local convolutional baselines remain difficult to beat. Boudjema et al. reported 99.71% WISDM accuracy with a 25K-parameter light CNN under a conventional frame-level evaluation protocol [17]. Seelwal et al. reported 87.85% accuracy for a standard CNN and 82.27% for SVM [18]. A recent Sensors article proposed redundancy-aware CNNs and pooling mechanisms for noisy mobile sensor data and achieved up to 96.84% weighted classification accuracy using histogram-based 2D encodings and novel pooling layers [16]. These results indicate that model comparison in WISDM must be interpreted together with evaluation protocol, split strategy, parameter count, and robustness objective.

The related literature therefore suggests three complementary paths. CNN-based methods emphasize local signal motifs and offer efficient architectures [6,7,17,19]. Recurrent and CNN-LSTM models introduce temporal memory for multimodal or longer-window signals [8,9,20]. Attention and state-space models seek broader temporal context, but their practical cost depends on architecture and implementation [10,11,12,13,14,15]. The present work combines a convolutional front end with a selective recurrent gate and attention and tests whether the resulting hybrid design improves recognition sufficiently to justify its additional cost. This is an empirical question rather than an assumed advantage over a compact CNN.

This paper develops and evaluates a Physics-Informed ConvFormer with State-Space Gating. Its design is motivated by three considerations. Acceleration magnitude is invariant to an ideal rotation of the measured vector, while acceleration-direction angles, a rolling gravity estimate, and gravity-relative components provide physically interpretable input quantities. These transformations do not guarantee that the complete representation is orientation-invariant or more discriminative. Local convolution supplies a temporal inductive bias for neighboring samples. A gated recurrent state provides a mechanism for retaining information within a window, although its utility and execution cost must be assessed experimentally. The gate has linear complexity in sequence length; the attention branch in the complete model retains quadratic sequence complexity. Here, physics-informed denotes physically motivated feature construction and convolution, not a physics-constrained training loss.

The main contributions are as follows: (1) a hybrid architecture combining deterministic acceleration feature construction, large-kernel depthwise convolution, causal state-space-style gating, and attention; (2) controlled comparisons with raw-input, module-ablated, Transformer-only, and compact CNN variants under four subject-level splits; and (3) an analysis of recognition accuracy, parameter count, and local inference cost that characterizes deployment-relevant trade-offs and identifies practical simplification options for resource-constrained use.

Figure 1 illustrates the overall architecture of the proposed Physics-Informed ConvFormer, from coordinate-decoupled accelerometer inputs to six-class HAR output.

## 2. Materials and Methods

### 2.1. Dataset and Windowing

Experiments used the public WISDM smartphone accelerometer data associated with the original WISDM study [1] and identified in the Data Availability Statement. The source publication reports that the WISDM project employed several Android smartphone models, including the Nexus One and HTC Hero (HTC Corporation, Taoyuan City, Taiwan) and the Motorola Backflip (Motorola Mobility LLC, Chicago, IL, USA) [1]; however, the public records do not identify the handset used for each participant or the manufacturer and model of the embedded accelerometer. The present study was a secondary analysis of this public dataset and used no additional data-collection equipment or materials. The analyzed file contains 36 user identifiers. Its records consist of x-, y-, and *z*-axis acceleration sampled at approximately 20 Hz during six daily activities, with the smartphone carried in a front pants pocket. Following the experimental code used in this study, the raw stream was segmented into windows of 128 samples with a stride of 64 samples. Windows were not allowed to cross either user boundaries or activity boundaries.

Table 1 shows the content composition and partitioning results of the WISDM dataset. The main run generated 10,922 training windows, 2036 validation windows, and 3234 test windows under a user-level split. This protocol is stricter than random frame-level splitting because unseen test users are not mixed into the training set. It therefore better reflects cross-user generalization but typically produces lower accuracy than overlapping-window random splits.

The use of a subject-level split changes how the results should be read. In a random window split, adjacent or near-adjacent windows from the same individual can appear in both training and test sets, allowing the model to learn user-specific gait amplitude, phone placement, and cadence. In a subject-level split, those cues are no longer shared with the test set. The measured accuracy therefore better approximates performance when the system is installed by a new user whose movement style was not present during training.

### 2.2. Coordinate Decoupling Layer

For each time sample, the original three acceleration channels were expanded to 13 input features. The layer retained raw x, y, and z values; computed vector magnitude r; converted the vector into spherical-coordinate features theta and phi; estimated a causal per-user gravity vector with a rolling mean; subtracted this gravity estimate to obtain dynamic x, y, and z components; and included the dynamic magnitude. The goal is not to discard the original axes, but to make the following network aware of both phone-coordinate motion and orientation-reduced physical quantities.

The derived quantities are intended to expose the acceleration magnitude, direction, and an approximate separation of static and dynamic components to the network. However, the rolling mean is a gravity estimate rather than an independently measured gravity vector, and the acceleration-direction angles are not direct measurements of body rotation. The ablation results reported in Section 3.3 show that the 13-channel representation did not improve average test accuracy over the raw three-channel variant. Its construction is physically motivated, but an empirical advantage in orientation robustness or cross-user generalization has not been established.

In implementation, all derived channels were computed before standardization, and the normalization statistics were fitted only on the training windows. This avoids validation or test leakage through global feature scaling. The gravity estimate was causal within each user sequence, so future samples were not used when forming the local static component. These details are important because apparently minor preprocessing choices can otherwise inflate WISDM performance.

### 2.3. Local Physics Convolution Layer

The local physics branch uses a large-kernel depthwise separable 1D convolution. The depthwise part captures channel-specific temporal motifs, while pointwise projection mixes channels with fewer parameters than a full convolution. The kernel size was set to 31 samples, corresponding to approximately 1.55 s at 20 Hz. This range is suitable for capturing local walking, jogging, and stair-motion periodicity while retaining the parameter efficiency of depthwise separable processing.

The large-kernel choice is physically motivated. A very small kernel may detect only instantaneous acceleration spikes, whereas activity recognition requires the shape of short movement cycles. Conversely, a very large convolutional kernel may blur transitions and add parameters unnecessarily. A kernel of 31 samples covers enough context to observe a substantial part of a walking or jogging cycle while remaining local compared with the full 128-sample window.

### 2.4. State-Space Gating and ConvFormer Blocks

Each ConvFormer block contains local convolution, a state-space-style gate, multi-head attention, and a residual MLP. The dependency-free PyTorch gate is inspired by content-dependent gating in Mamba [13] but is not a full Mamba implementation. Each embedded time step is projected into a signal, an input gate, and an output gate. A causal depthwise convolution processes the signal, and a learned channel-wise decay controls recurrent state retention. The recurrence is linear in sequence length and is executed by an explicit Python (Version 3.12.13) loop over all 128 time steps in each of four blocks. This algorithmic complexity does not guarantee low measured latency; the runtime consequences are evaluated in Section 3.3 and Section 4.

The attention branch provides global mixing after local convolution and recurrent gating. The design combines different temporal operations, but whether this combination improves the accuracy-cost trade-off relative to a compact CNN is determined by the ablation results rather than by the architecture alone.

In the implemented forward pass, local convolution, State-Space Gating, attention, and the MLP are applied in that order. Each sublayer receives a layer-normalized representation and has its own residual addition. The recurrent state is reset for each input window; the current model does not maintain memory across consecutive windows. Accordingly, the experiments evaluate within-window temporal processing, not continuous long-duration streaming memory.

Figure 2 presents a detailed layer-level view of the implemented network. It shows how the Coordinate Decoupling Layer, Local Physics Convolution Layer, and State-Space Gating and ConvFormer Blocks transform a raw B × 128 × 3 accelerometer window into a six-class HAR prediction.

### 2.5. Training Protocol and Metrics

Table 2 summarizes the model configuration and training protocol. Training used class-weighted cross-entropy, AdamW, weight decay, dropout, gradient clipping, a cosine learning-rate schedule, and early stopping based on validation accuracy. The evaluation follows explicit train-validation-test separation and repeated comparisons [20,21]. The main experiment allowed 40 epochs and stopped at epoch 22. The ablation experiments allowed 20 epochs with patience 5, batch size 256, and subject-level split seeds 42, 123, 2026, and 3407. These seeds change subject assignment and stochastic training, so the four-run variability does not isolate either source. Accuracy, precision, recall, F1-score, parameter count, estimated FP32 parameter storage, training time, and local forward-pass latency per window were recorded.

Latency was measured by the ablation script using an already constructed input tensor of shape 1 × 128 × C, with the model in evaluation mode and gradient recording disabled. Five warm-up forward passes preceded 30 timed passes; latency is the mean forward-pass time per window. The corresponding four-run measurements are reported with the ablation results in Section 3.3. Feature construction and other preprocessing were outside the timed region. Because no on-device profiling was performed and processor, thread count, and runtime metadata were not retained in the summary files, the values are interpreted as implementation-specific local comparisons rather than a reproducible smartphone deployment benchmark.

## 3. Results

### 3.1. Main Training Behavior

The convergence behavior of the main experiment is shown in Figure 3, where the validation curve stabilizes after the early learning stage while the training curve continues to approach saturation.

The main run reached a best validation accuracy of 95.63% and a test accuracy of 83.92%. Validation accuracy increased rapidly during the first epochs and stabilized near 95%, while training accuracy approached 100%. The train-validation gap indicates that the model fits the training windows strongly and that cross-user generalization remains the primary challenge.

### 3.2. Class-Level Test Results

The class-level precision, recall, and F1-score values are reported in Table 3, together with overall accuracy, macro-average metrics, and support-weighted metrics. These results provide the basis for interpreting both aggregate performance and activity-specific weaknesses.

As shown in Figure 4, Jogging was the most reliable class, with an F1-score of 95.71%, followed by Downstairs and Walking. The per-class F1-score distribution indicates that high-amplitude dynamic activities were generally easier to distinguish than static or transition-related activities. Standing achieved 100% recall, but its precision was lower because a portion of Sitting windows were predicted as Standing. This Sitting–Standing ambiguity is further confirmed by the confusion matrix in Figure 5. Upstairs was the weakest class, mainly because many Walking windows were confused with Upstairs and several stair windows were close to other dynamic activities, as also observed in Figure 5. References [16,17,18] provide WISDM-based comparison context, but differences in preprocessing and evaluation protocol prevent attributing the present class-level errors directly to those studies.

### 3.3. Ablation Study Across Four Subject-Level Splits

Table 4 reports mean and sample standard deviation across four subject-level splits. The comparisons assess both predictive performance and computational cost; an improvement over an ablated hybrid variant does not imply superiority over the separate CNN baseline.

The full model achieved 88.86 ± 6.16% mean test accuracy, compared with 89.66 ± 5.56% for the CNN baseline. Thus, the CNN exceeded the full model by 0.80 percentage points on average. The full model required 588,006 parameters versus 125,766 for the CNN, approximately 4.68 times as many, and had a mean measured latency of 21.54 ms versus 0.45 ms, approximately 47.87 times higher using the rounded values in Table 4. The full model exceeded the CNN in two splits and underperformed it in two. Accordingly, the controlled mean comparison favors the CNN baseline on both accuracy and measured efficiency. Within the hybrid architecture, removing local convolution or attention reduced mean accuracy by 3.99 or 3.06 percentage points, respectively. These module-level results clarify which components contribute within the hybrid backbone and should be interpreted separately from the full-model comparison with the compact CNN.

The raw_xyz_full variant achieved 89.54 ± 5.07% mean test accuracy, 0.68 percentage points above the full 13-channel representation. The full model exceeded raw_xyz_full for seeds 42 and 3407, but underperformed it for seeds 123 and 2026. The derived channels are deterministic functions of the same accelerometer measurements, not additional independent sensor observations. Consequently, the tested splits show no consistent advantage from fixed coordinate decoupling. Redundant features, the approximate gravity estimate, and the dependence of directional features on phone orientation are possible explanations, but their individual effects were not isolated by these experiments.

The no_ssm variant achieved 88.64 ± 6.09% mean test accuracy. The full model’s average gain was approximately 0.22 percentage points, with an improvement in two splits and a reduction in two. Removing the gate reduced mean latency from 21.54 to 3.80 ms, approximately 82.36%, and parameter count from 588,006 to 435,558, approximately 25.93%. Thus, the full model was approximately 5.67 times slower for a small, split-dependent accuracy difference. For latency-sensitive use, the no_ssm variant offers a lower-cost hybrid alternative under the tested conditions. All differences are descriptive: four subject-level splits, with possible overlap in held-out users and variation in stochastic training, do not establish statistical significance or performance for every individual user.

Figure 6 displays the mean test accuracies in Table 4. It should be read together with the uncertainty, parameter counts, and latency values: local convolution and attention contributed within the hybrid backbone, but the CNN and raw-input variants had higher mean accuracy than the full model.

### 3.4. Comparison with Previous Studies

Table 5 summarizes four reference studies and the present study, including model type, representation, and evaluation protocol.

Reported WISDM accuracy varies across sliding-window, random train/test, and subject-level evaluations [16,17,18]. Differences in held-out subjects, overlapping windows, training settings, and metrics prevent these published values from being treated as a direct model ranking. The same-split CNN baseline in Table 4 provides the relevant controlled comparison. In this comparison, the CNN achieved slightly higher mean accuracy with lower measured local latency. These protocol-dependent literature values are visualized as contextual information in Figure 7.

Figure 7 visualizes published accuracy values alongside the present validation and test results. Validation accuracy and test accuracy answer different questions and must not be treated as interchangeable. BabyMamba-HAR [15] also investigates state-space modeling, but its deployment evidence does not validate the present dependency-free recurrent implementation. The present study evaluates a WISDM hybrid architecture and its component trade-offs; it does not reproduce the embedded deployment experiments of that reference.

## 4. Discussion

The principal finding is a clearly quantified accuracy-cost trade-off. As shown in Table 4, the compact CNN obtained higher mean test accuracy (89.66% versus 88.86%), while the full model required approximately 4.68 times as many parameters and had approximately 47.87 times the local forward-pass latency. Accordingly, the practical contribution of the full hybrid model lies not in claiming baseline superiority, but in the controlled assessment of how local convolution, State-Space Gating, and attention affect predictive performance and computational cost. The 588,006-parameter model has approximately 2.24 MB of FP32 parameter storage, excluding activation memory and runtime overhead. The model is compact in parameter storage, while the latency measurements provide a concrete basis for targeted optimization before on-device evaluation.

The benefits of local convolution and attention are conditional on the remaining hybrid backbone. Removing them reduced mean accuracy by 3.99 and 3.06 percentage points, respectively (Table 4 and Figure 6). This supports retaining these operations within the tested hybrid configuration, but it does not establish that large-kernel convolution is optimal, that physical features are necessary, or that recurrent memory improves on a simpler CNN. The Transformer-only result likewise provides a comparison with that particular implementation, not a general conclusion about all attention-based HAR models. The CNN itself provides a local temporal inductive bias and remains a strong baseline under the same subject-level evaluation.

The coordinate-decoupled representation did not provide an average recognition advantage. The raw-input hybrid obtained 89.54% versus 88.86% for the full representation (Table 4), and their relative order changed across splits. Expanding three channels into thirteen re-expresses existing measurements; it does not add information from an independent sensor. Although this representation makes some physical quantities explicit, the backbone can also learn transformations of the raw channels. The causal rolling mean may include movement components, and acceleration-direction angles do not by themselves resolve phone placement variability. These are plausible sources of redundancy or estimation error, not demonstrated explanations of the observed difference. The results therefore support keeping raw input as a strong alternative and treating fixed feature expansion as an unconfirmed design choice. Learnable feature selection or orientation adaptation would require new experiments before any benefit could be asserted.

The SSM ablation indicates a limited accuracy-cost benefit under the current implementation. The approximately 0.22-percentage-point mean gain over no_ssm was accompanied by an increase from 3.80 to 21.54 ms per window (Table 4), and the full model achieved higher accuracy in two of four splits. The source code executes 128 sequential recurrent updates per block in a Python loop across four blocks. Repeated small tensor operations and sequential state dependencies are a plausible explanation for the measured overhead, but the benchmark does not isolate the time spent in each operation. Linear sequence complexity describes scaling and does not imply faster execution than parallel convolutions at this window length. In addition, the complete model retains attention, and its recurrent state is reset between windows. Longer-context benefits and continuous streaming memory were not tested. Under the measured setting, no_ssm is a lower-cost hybrid alternative, while the compact CNN offers the strongest observed overall accuracy-cost trade-off. Optimized recurrent kernels, longer windows, or continuous state retention may alter this comparison and should be examined in future work.

The class-level results in Table 3 and Figure 4 and Figure 5 also support the interpretation of overall accuracy. Upstairs had an F1-score of 0.5602, while Sitting recall was 0.5422. Table 3 reports a macro F1-score of 0.7879 and a support-weighted F1-score of 0.8485, while Figure 5 visualizes the corresponding error distribution. These results show why the 83.92% overall accuracy should not be read as uniform reliability across activities. The observed Walking–Upstairs and Sitting–Standing confusions are established by the predictions; their attribution to missing gyroscope measurements or phone orientation remains a hypothesis. This accelerometer-only experiment did not test sensor fusion, so the effect of additional inertial channels would require separate evaluation [8,22].

Subject-level separation prevents test users from contributing training windows, but four group-based splits do not establish reliable performance for every unseen individual. The full model’s split accuracies ranged from 83.80% to 96.94%, with a sample standard deviation of 6.16 percentage points (Table 4). This variability reflects changes in held-out subject composition together with stochastic training, and is not a per-user performance distribution. A leave-one-subject-out evaluation and repeated training on fixed subject partitions would be needed to separate these effects. Table 5 and Figure 7 provide literature context only, because protocol and metric differences prevent direct cross-study ranking.

The data were collected using smartphones, but the model was not deployed or benchmarked on a smartphone in this study. The latency results are local CPU forward-pass measurements on one preconstructed window, not end-to-end mobile measurements. They exclude physical-feature computation, normalization, window buffering, sensor acquisition, and mobile runtime overhead. The benchmark used evaluation mode, disabled gradient recording, five warm-up passes, and 30 timed passes at batch size one. Its results characterize this implementation and local environment rather than device-specific latency, battery consumption, peak memory, or sustained mobile operation. Accordingly, the term smartphone-based refers to the origin of the accelerometer recordings. Direct on-device validation of latency, energy, memory, and sustained operation is a clear next step.

## 5. Conclusions

This study evaluated a Physics-Informed ConvFormer with State-Space Gating on smartphone-recorded WISDM accelerometer data under subject-level separation. The main run achieved 95.63% best validation accuracy and 83.92% test accuracy; the four-split ablation study yielded 88.86 ± 6.16% mean test accuracy. Local convolution and attention contributed within the tested hybrid backbone, while the controlled comparison favored the compact CNN in mean accuracy and measured efficiency. Fixed 13-channel feature construction did not improve average accuracy over raw three-channel input. The SSM gate produced a small average accuracy difference at higher latency, identifying no_ssm as a practical simplification option for latency-sensitive settings. Overall, the study provides transparent component-level evidence on hybrid temporal modeling and a concrete roadmap for mobile optimization. Direct smartphone profiling, broader subject-level validation, and independent assessment of orientation robustness and longer-context benefits are the next steps toward establishing deployment readiness.

## Figures and Tables

**Figure 1:**
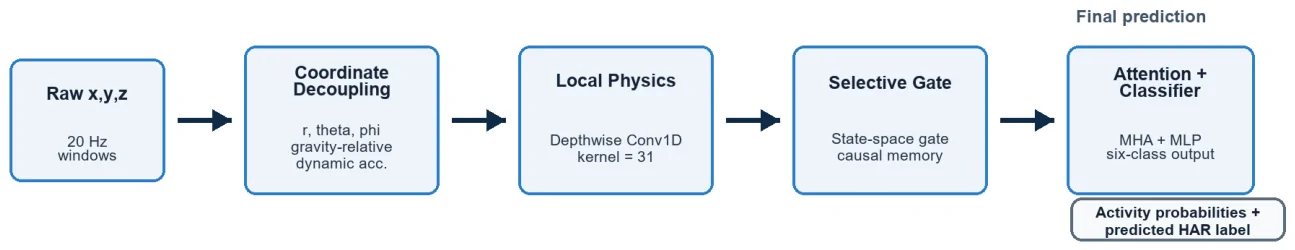
Overall architecture of the proposed Physics-Informed ConvFormer.

**Figure 2:**
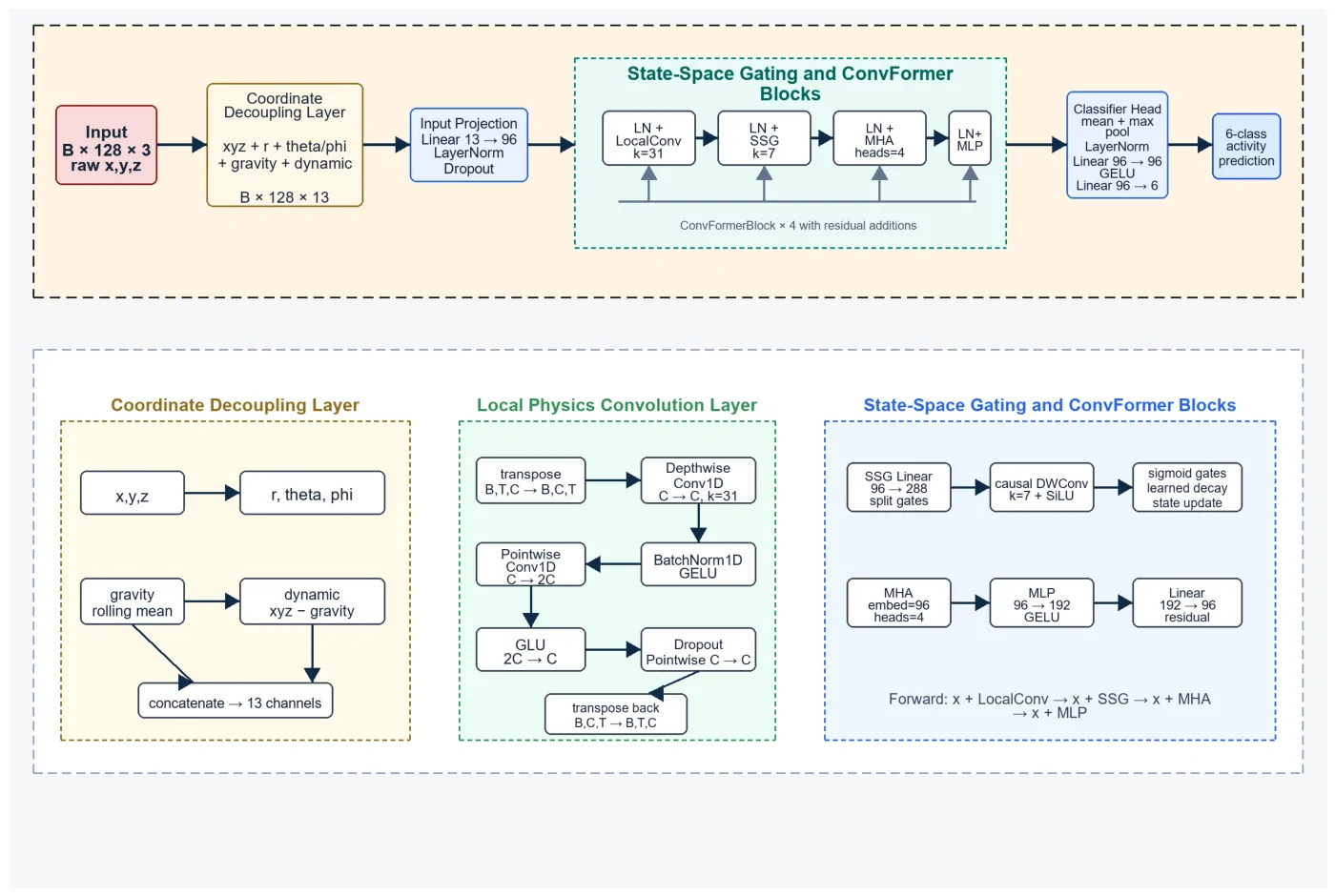
Detailed layer-level architecture of the Physics-Informed ConvFormer, including the Coordinate Decoupling Layer, Local Physics Convolution Layer, State-Space Gating and ConvFormer Blocks, classifier head, and six-class HAR output.

**Figure 3:**
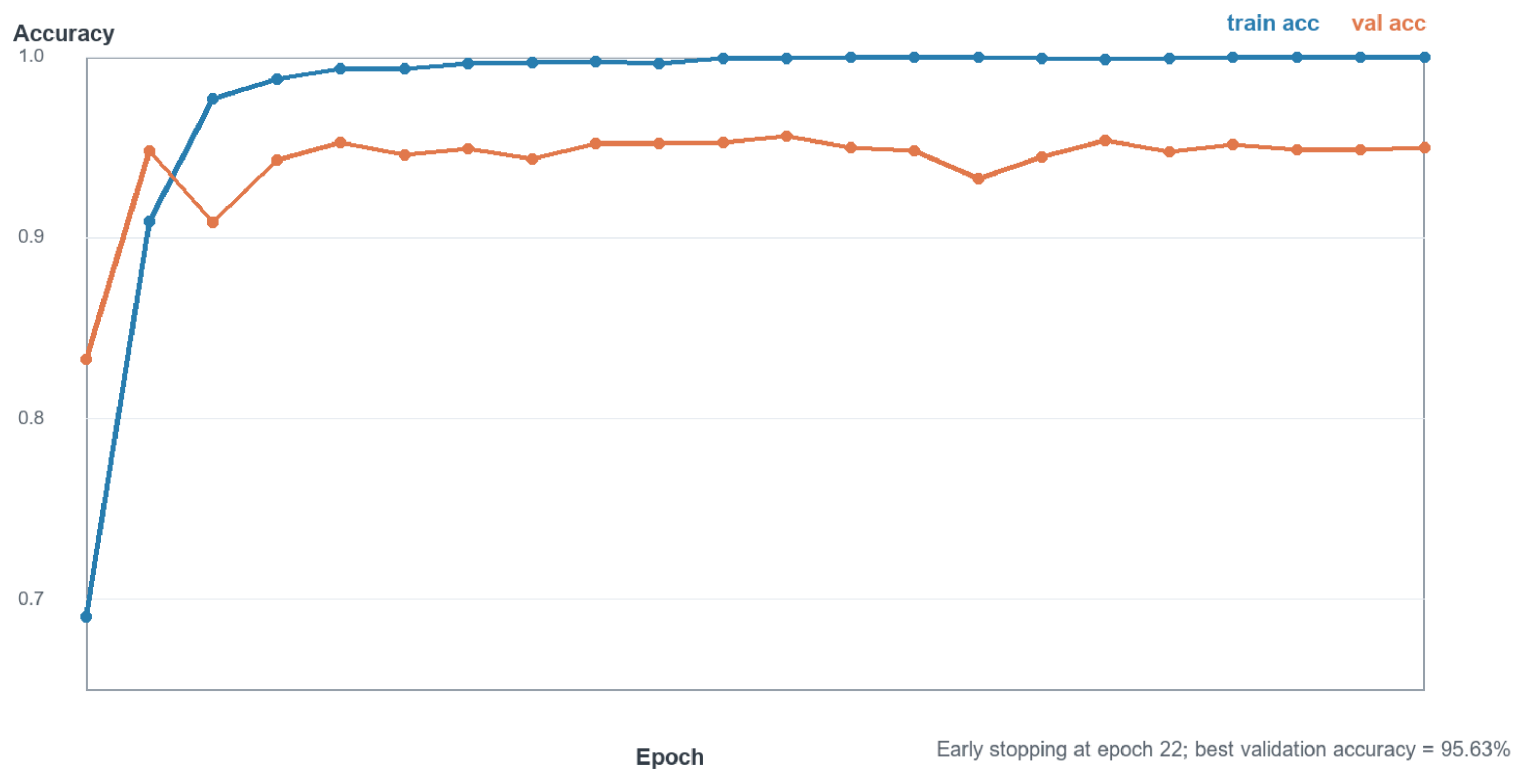
Training and validation accuracy from the main experiment.

**Figure 4:**
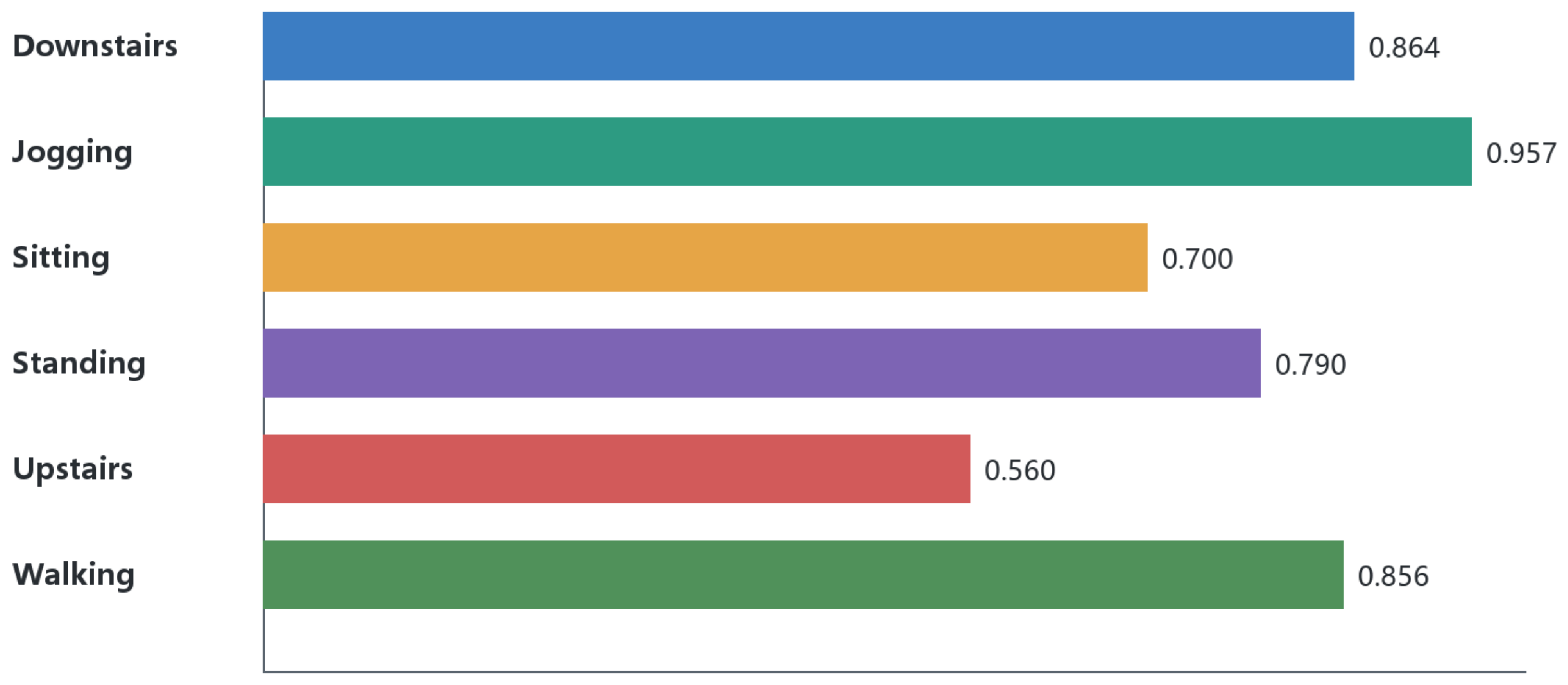
Per-class F1-score distribution on the main subject-level test split. The highest F1-score is obtained for Jogging (0.957), whereas the weakest class is Upstairs (0.560), suggesting stair-direction ambiguity.

**Figure 5:**
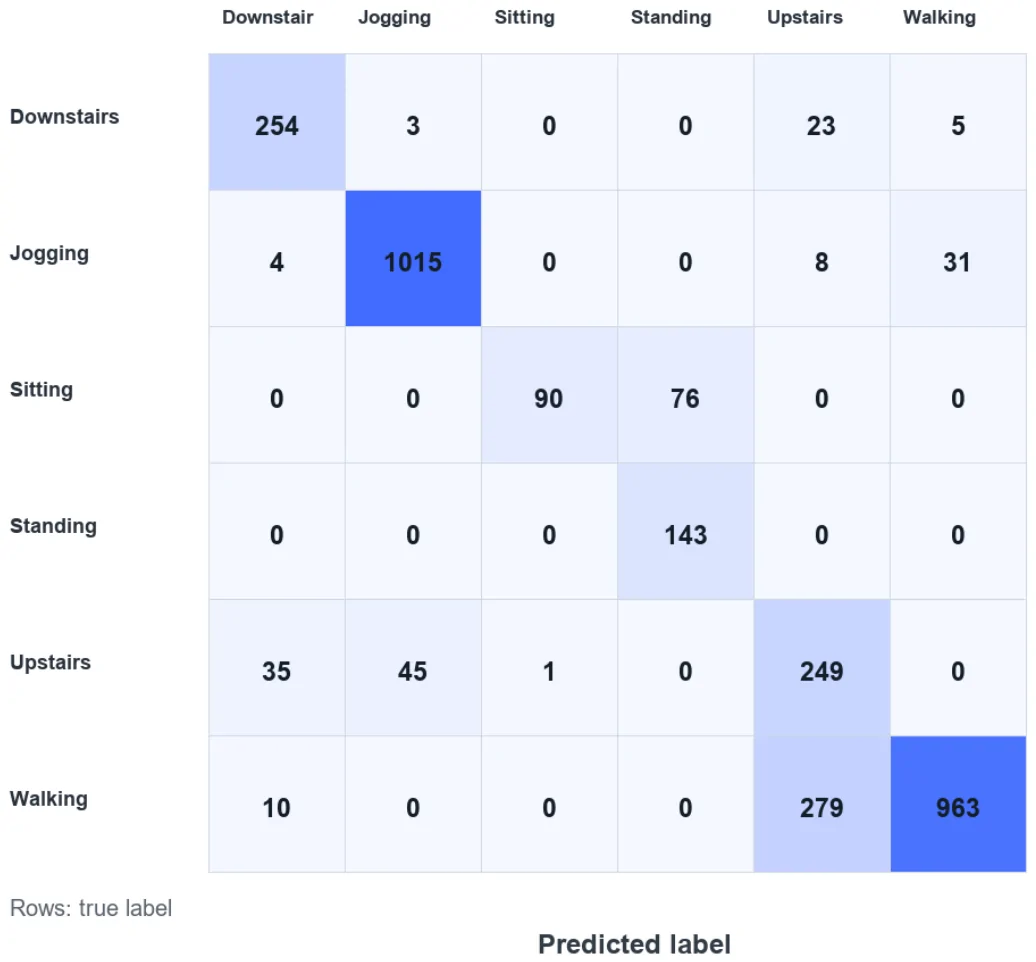
Confusion matrix for the main subject-level test split.

**Figure 6:**
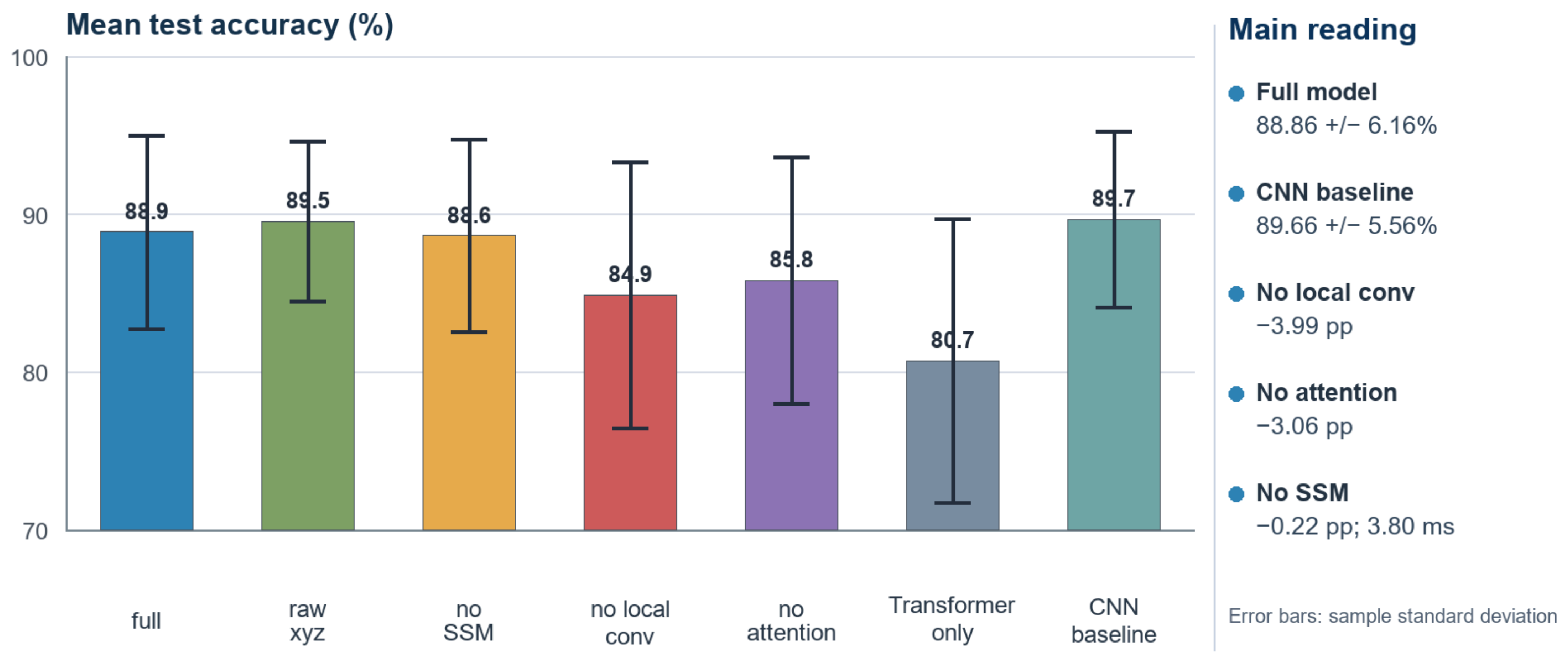
Mean test accuracy across four subject-level splits for the full model and comparison variants. Accuracy differences alone do not establish an advantage in computational efficiency.

**Figure 7:**
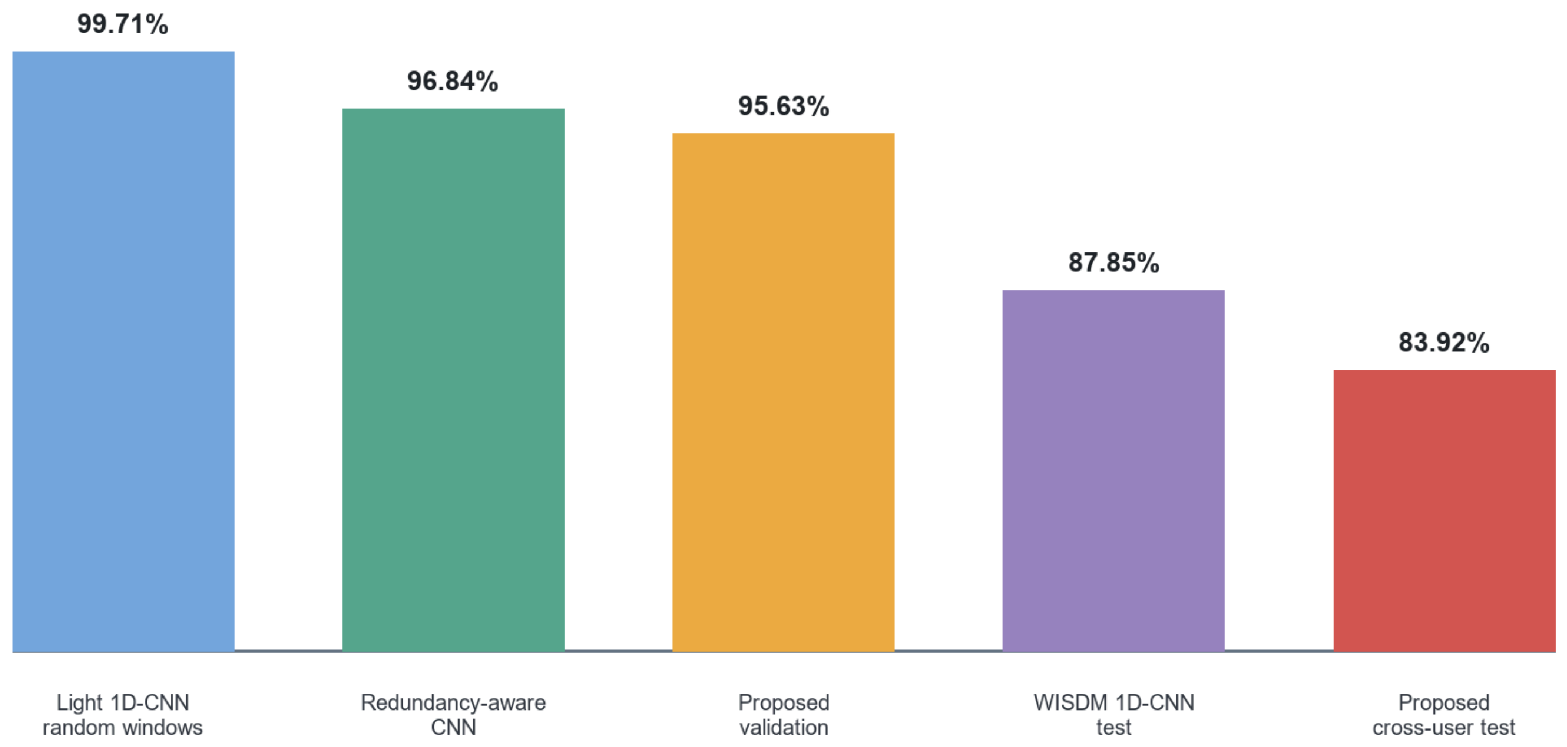
Contextual comparison of reported WISDM-related accuracies under different evaluation protocols. These values do not constitute a controlled performance ranking.

**Table 1:** Dataset, segmentation, and subject-level split settings.

Item	Description
Dataset	WISDM v1.1 smartphone accelerometer
Subjects	36 users
Sensor	Tri-axial smartphone accelerometer
Sampling rate	Approximately 20 Hz
Activities	Downstairs, Jogging, Sitting, Standing, Upstairs, Walking
Window length	128 samples, approximately 6.4 s
Window stride	64 samples, 50% overlap
Main split	User-level split: 10,922 train, 2036 validation, 3234 test windows

**Table 2:** Model configuration and experimental protocol.

Component	Setting
Input representation	13 channels: raw axes, magnitude, angular, gravity-relative, and dynamic features
Embedding/backbone	Physics-Informed ConvFormer with local depthwise convolution, state-space gate, attention, and MLP
Main model size	588,006 parameters; approximately 2.24 MB FP32 parameter storage, excluding activations and runtime memory
Main training objective	Cross-entropy classification over six WISDM activity classes
Optimizer and regularization	AdamW, weight decay, dropout, gradient clipping, and early stopping
Main run budget	Maximum 40 epochs; stopped at epoch 22
Ablation budget	Maximum 20 epochs; patience 5; batch size 256
Ablation seeds	42, 123, 2026, and 3407
Reported metrics	Accuracy, precision, recall, F1-score, parameter count, model size, latency, and training time

**Table 3:** Main test-set classification report for six WISDM activities, including aggregate metrics.

Activity	Precision	Recall	F1-Score	Support
Downstairs	0.8383	0.8912	0.8639	285
Jogging	0.9548	0.9594	0.9571	1058
Sitting	0.9890	0.5422	0.7004	166
Standing	0.6530	1.0000	0.7901	143
Upstairs	0.4454	0.7545	0.5602	330
Walking	0.9640	0.7692	0.8556	1252
Overall accuracy	-	-	0.8392	3234
Macro average	0.8074	0.8194	0.7879	3234
Weighted average	0.8845	0.8392	0.8485	3234

**Table 4:** Mean ± sample standard deviation across four random subject-level splits. Latency measures local forward-pass time per preconstructed window; MB denotes estimated FP32 parameter storage.

Variant	Input	Params	MB	Val Acc (%)	Test Acc (%)	Latency (ms)
full	13	588,006	2.24	87.77 ± 6.60	88.86 ± 6.16	21.54 ± 3.29
raw_xyz_full	3	587,046	2.24	91.03 ± 8.47	89.54 ± 5.07	18.60 ± 1.38
no_ssm	13	435,558	1.66	90.58 ± 6.20	88.64 ± 6.09	3.80 ± 0.11
no_local_conv	13	464,742	1.77	87.89 ± 8.60	84.87 ± 8.42	16.86 ± 2.11
no_attention	13	588,006	2.24	89.87 ± 8.26	85.80 ± 7.79	18.34 ± 1.14
transformer_only	13	310,758	1.19	89.27 ± 4.44	80.69 ± 9.00	1.62 ± 0.13
cnn_baseline	3	125,766	0.48	89.82 ± 8.67	89.66 ± 5.56	0.45 ± 0.02

**Table 5:** Comparison with four recent HAR reference studies and the present study.

Study	Model	Protocol	Reported Result	Relevance
Seelwal et al. [18]	Standard 1D-CNN on WISDM	Train/test evaluation reported; no subject-disjoint protocol identified	87.85% test accuracy	CNN reference on WISDM; direct ranking is limited by protocol differences
Boudjema et al. [17]	Light CNN	70% training and 30% evaluation sample split; no subject-disjoint protocol reported	99.71% accuracy, 25K params	Strong compact CNN result under a different evaluation protocol
Ameen and Aminifar [16]	Redundancy-aware CNN + histogram encoding + novel pooling	WISDM clean/noisy evaluation	96.84% weighted accuracy	Robustness-oriented CNN with explicit noise experiments
BabyMamba-HAR [15]	Selective SSM/Mamba TinyML HAR	Eight benchmarks, five seeds	86.52% average F1, approx. 27K params	Direct support for efficient state-space HAR on constrained devices
This study	Physics-Informed ConvFormer + SSM gate	Subject-level WISDM splits	83.92% main test; 88.86 ± 6.16% across four splits	Hybrid physics-aware local convolution, causal memory, and attention; stricter cross-user evaluation

## Data Availability

The dataset analyzed in this study is publicly accessible in the Human Activity Recognition dataset on Kaggle at https://www.kaggle.com/datasets/die9origephit/human-activity-recognition (accessed on 7 July 2026). The code used for analysis is available from the corresponding author upon reasonable request.
